# Does feedback on daily activity level from a Smart watch during inpatient stroke rehabilitation increase physical activity levels? Study protocol for a randomized controlled trial

**DOI:** 10.1186/s13063-018-2476-z

**Published:** 2018-03-09

**Authors:** Yun Dong, Dax Steins, Shanbin Sun, Fei Li, James D. Amor, Christopher J. James, Zhidao Xia, Helen Dawes, Hooshang Izadi, Yi Cao, Derick T. Wade, Smart watch activity feedback trial committee (SWAFT), Yuanfeng Peng, Jingjing Xue, Xiaoli Guo, Xuesong Xie, Na Zuo, Xinkui Gao, Lingzhi Wu, Peifang Li, Ying Wang, Chong Chen, Peiyang Sun, Jinji Wang, Feifei Wang, Panfu Hao, Weiwei Wu, Yubao Gao, Xiaoli Sun, Haiyang Wu, Yujie Yang

**Affiliations:** 1 Rehabilitation Centre, the Second Affiliated Hospital of Anhui University of Traditional Chinese Medicine, Hefei, Anhui Province China; 2 Movement Science Group, Centre for Rehabilitation, Oxford Institute of Nursing and Allied Health Research, Oxford Brookes University, Oxford, UK; 3 School of Engineering, University of Warwick, Coventry, UK

**Keywords:** Stroke, Physical activity, Technology, Goal setting, Feedback

## Abstract

**Background:**

Practicing activities improves recovery after stroke, but many people in hospital do little activity. Feedback on activity using an accelerometer is a potential method to increase activity in hospital inpatients. This study’s goal is to investigate the effect of feedback, enabled by a Smart watch, on daily physical activity levels during inpatient stroke rehabilitation and the short-term effects on simple functional activities, primarily mobility.

**Methods/design:**

A randomized controlled trial will be undertaken within the stroke rehabilitation wards of the Second Affiliated hospital of Anhui University of Traditional Chinese Medicine, Hefei, China. The study participants will be stroke survivors who meet inclusion criteria for the study, primarily: able to participate, no more than 4 months after stroke and walking independently before stroke. Participants will all receive standard local rehabilitation and will be randomly assigned either to receive regular feedback about activity levels, relative to a daily goal tailored by the smart watch over five time periods throughout a working day, or to no feedback, but still wearing the Smart watch. The intervention will last up to 3 weeks, ending sooner if discharged. The data to be collected in all participants include measures of daily activity (Smart watch measure); mobility (Rivermead Mobility Index and 10-metre walking time); independence in personal care (Barthel Activities of Daily Living (ADL) Index); overall activities (the World Health Organization (WHO) Disability Assessment Scale, 12-item version); and quality of life (the Euro-Qol 5L5D). Data will be collected by assessors blinded to allocation of the intervention at baseline, 3 weeks or at discharge (whichever is the sooner); and a reduced data set will be collected at 12 weeks by telephone interview. The primary outcome will be change in daily accelerometer activity scores. Secondary outcomes are compliance and adherence to wearing the watch, and changes in mobility, independence in personal care activities, and health-related quality of life.

**Discussion:**

This project is being implemented in a large city hospital with limited resources and limited research experience. There has been a pilot feasibility study using the Smart watch, which highlighted some areas needing change and these are incorporated in this protocol.

**Trial registration:**

ClinicalTrials.gov, NCT02587585. Registered on 30 September 2015. Chinese Clinical Trial Registry, ChiCTR-IOR-15007179. Registered on 8 August 2015.

**Electronic supplementary material:**

The online version of this article (10.1186/s13063-018-2476-z) contains supplementary material, which is available to authorized users.

## Background

After stroke, practicing an activity and being active helps increase the speed and/or extent of recovery [1] but it is well-established that most inpatients in stroke rehabilitation settings have low levels of activity [2–4]. Increasing patients’ self-generated activity is possible through reorganizing care [5, 6] and could potentially play an important role in increasing independence.

Another way to increase activity and therefore to improve recovery of independence is to provide tailored feedback on activity and on progress towards goals. This could increase motivation, self-efficacy, and self-generated activity. Accelerometers can be used to record activity [2], and they can also be used to provide feedback; use of daily data by therapists can increase time spent walking [7]. There are several studies investigating the effect of feedback about physical activity from accelerometers to individuals. Some show no effect [8, 9] but some show beneficial effects [7, 10]. Benefits seem more likely if feedback is supported by other motivational support [11]. Most of these studies were published after the design of this project; there are many other study protocols published but not yet completed.

The primary objective of this study is to determine whether patient feedback about the amount of activity undertaken compared to their activity the previous day, provided at fixed time points during the day, will increase patient activity. We hypothesise that, compared to a control group who do not receive feedback from a Smart watch, those who receive feedback on daily activity will have increased physical activity levels, as measured by the activity scores, with no harms. We also hypothesize that this increased activity during inpatient stroke rehabilitation will result in improved mobility, and possibly cognition, arm function, independence in daily activities, and health-related quality of life. A third objective is to investigate the feasibility of setting up large-scale trials in a research-naïve setting. This protocol follows an initial pilot and feasibility study. (Lawrie S, Gong Y, Steins D, Xia Z, Esser P, Sun S, Li F, Amor J, James C, Izadi H, Chao Y, Wade DT, Dawes H: Augmented Feedback of Daily Activity on Physical Activity Levels after Acute Stroke: a pilot randomised controlled feasibility study, in preparation).

## Methods

This study was originally registered with ClinicalTrials.gov on 30 September 2015 (NCT02587585). The original registration was for both the initial pilot and feasibility phase, now completed (submitted for publication), and this phase, which has been adjusted from the original in the light of the pilot phase. The trial was also registered with the Chinese Clinical Trial Registry (http://www.chictr.org.cn/showprojen.aspx?proj=12091) on 8 August 2015 (ChiCTR-IOR-15007179). As part of this registration it was also considered by the Chinese National Ethics committee and given a favourable opinion; the certificate is within the registration details, and has reference number ChiECRCT-20150034 (see http://www.chictr.org.cn/uploads/file/20151009090354.JPG). All research will be in compliance with the Helsinki Declarations and the Research Governance Framework for Health and Social Care. Informed consent will be obtained from all participants before involvement in the study.

The trial sponsor is The Second Affiliated Hospital of Anhui University of Traditional Chinese Medicine, Hefei, Anhui Province, China. The public contact is Zhidao Xia, the scientific contact is Derick Wade. This protocol (6 February 2017) has been amended from the original registered protocol (30 September 2015) in the light of the feasibility study; data will not be collected at 6 months because this is not practical. This study started after the feasibility phase, on 20 April 2016 and by 6 February 2017 (date of original submission) had recruited 88 of a target of 200 people.

The study team (ZX, HD, YD) confer by video-phone conference every 2–4 weeks. The local committee is the Smart Watch Activity Feedback Trial Committee (SWAFT), whose members are given in the title page. They meet to resolve any local practical problems. There is no additional data monitoring committee because harmful effects are considered improbable and the study will be short. The primary specific funding body did not undertake an external peer review of the protocol.

### Design

This study is an efficacy, single-blind, randomized controlled trial with the assessor blinded to the group allocation. Participants cannot be blinded to group allocation and ward staff will not be. The intervention being studied is for all patients to wear a Smart watch throughout a normal working day (0800–1700 hours) from Monday to Friday for up to 15 days. They will be randomly assigned, using concealed allocation, to one of two groups where the watch gives:

- Feedback on amount of physical activity (movement) undertaken, two hourly for the first 8 hours and then for the last 1–2 hours (dependent on the battery life) or
- No feedback about the physical activity levels during a 3-week intervention period.

All other interventions (medication, physical therapy) will be kept to their normal routine.

**Fig. 1:**
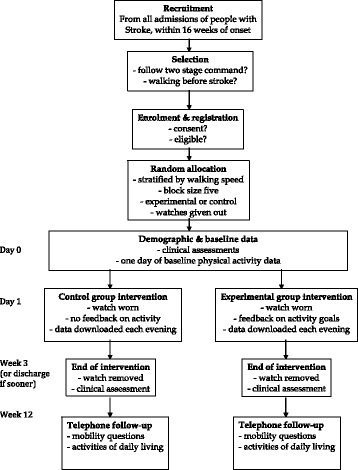
Consolidated Standards of Reporting Trials (CONSORT)-style flow diagram

The flow of the study is shown in Fig. 1. Patients will be recruited from all admissions to the rehabilitation wards in the Second Affiliated Hospital of Anhui University of Traditional Chinese Medicine, a large urban hospital. Only patients admitted for rehabilitation after a recent stroke will be approached. The procedure used needs to be compatible with normal clinical practice.

Two questions will be used for initial screening:

1. Can the patient follow a two-stage command, judged clinically?
2. Was the patient walking without help from another person before this stroke?

All subjects who answer “yes” to both questions are eligible and will be entered into the consent and selection process. An investigator will explain the consent form, and allow the person ample time to read through the consent form and ask questions. If they agree, they will acknowledge consent by signing the form. All potential participants will have up to 24 hours to decide whether they want to partake in this study. All actual participants will have given informed consent, signed by them or by an independent witness if necessary, before they are registered.

Patients will be eligible if they:

- Are aged 40–75 years
- Had onset of stroke less than 4 months ago
- Are being admitted for rehabilitation for the first time
- Were able to walk at least 10 m prior to stroke, without help of another person; use of equipment allowed
- Have sufficient cognition to participate in the study and testing procedures (clinically judged, because there are no valid short cognitive measures to assess ability to consent and participate)
- Can follow a 2-stage command (e.g. pick up an object, put it on the table)
- Have sufficient visual function to see the watch feedback (clinically judged)
- Have the capacity to consent and then give consent to participate in the study

Entry may be delayed after admission if a person satisfied all other criteria but was not yet able to follow a 2-stage command. In this case, 3-day follow up will be performed, and the patient will be eligible if they then meet that criterion. This was added to allow for patients transferred within 1 or 2 weeks of a stroke, and patients who become confused by the move from another hospital. Patients admitted a second time within the study period will be excluded.

Participants will be randomly assigned to one of two groups: (1) feedback of activity levels; or (2) no feedback of activity levels (i.e. control group). A block stratified randomization approach with two strata will be used, based on walking speed at the time of recruitment. A threshold of 0.42 m/s [12] will be used to define two groups: slow walkers (< 0.42 m/s) unlikely to walk in the community, and moderate-fast walkers (> 0.42 m/s). People unable to walk at the time of recruitment will be classified as slow walkers. The block size will be 5.

Once recruited, the recruiting doctor will inform the research office personnel, who are not blinded, and they will then undertake allocation. Allocation will be performed using a computer program in Microsoft Excel to allocate groups and generate numbers for each stratum (slow walkers or not slow walkers); the allocation will be made by the research office staff after registration. The research office will then notify the ward about the patient’s allocation by telephone. The researchers who will be performing all assessments will remain blinded to group assignment throughout the study.

The participants’ rehabilitation programmes will continue as normal, with no change in relation to this study. Therapists will not specifically be informed about a participant’s group, but may learn this. However therapists will not specifically encourage use of the feedback (or the opposite).

#### Intervention - the China Monitoring (CHMON) feedback system

The CHMON system utilized for the project is a ZGPAX S8 [13]. The S8 is a full android Smart watch encapsulated in a wrist-wearable unit. As such it is capable of running apps written for it and operating in a stand-alone manner. The hardware specifications for the ZGPAX S8 are shown in Table 1.

**Table 1:** Hardware specifications for the ZGPAX S8 android Smart watch

	Hardware specification
Size	58 × 42.5 × 13 mm
Weight	67 g
Screen	40 mm capacitive touchscreen
Battery	3.7 V/470mAh Li-ion (rechargeable)
CPU	MTK6572 Dual Core 1.2 GHz
Memory	12 GB total
RAM	512 MB
Android version	4.4.2
Sensors	GPS, accelerometer
Connectivity	WiFi, Bluetooth, 2G, 3G
Waterproof	No

*CPU* central processing unit, *RAM* random access memory

**Fig. 2:**
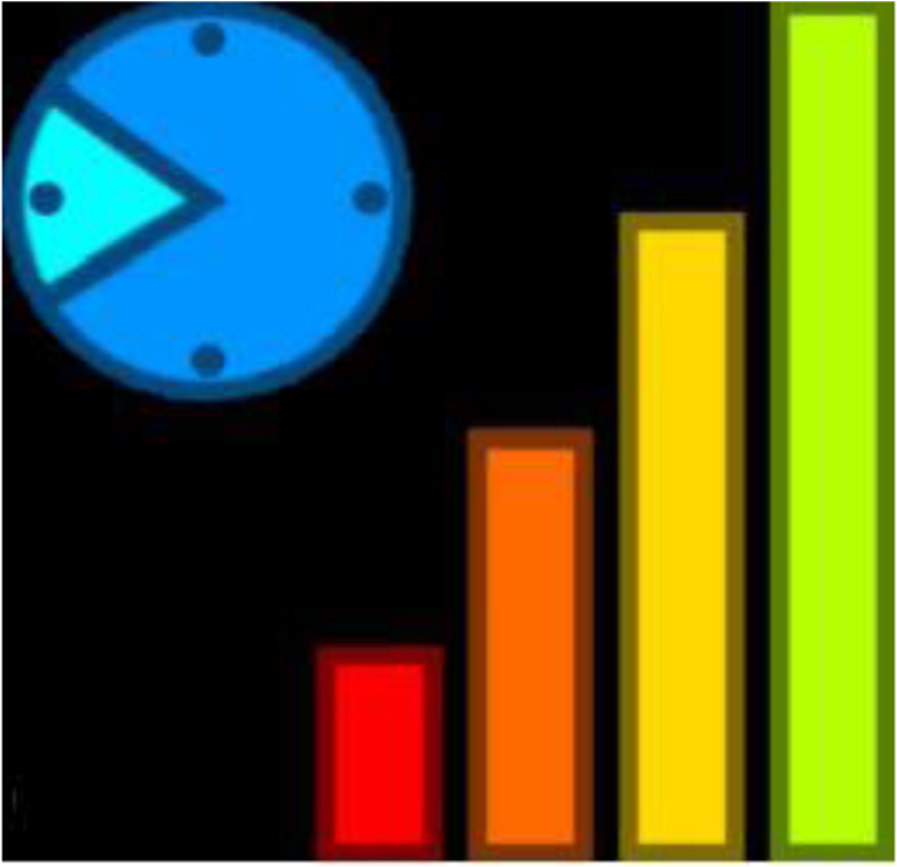
The watch face: blue clock icon showing current 2-hour window and red-green bars showing activity feedback (one group only)

The CHMON system is used both as a data gathering platform and as a feedback device to the patient in the experimental group (Fig. 2). The patient wears the device for 9 hours a day. In the intervention group, the watch will divide elapsed time into four periods of 2 hours, and one of 1 hour. In the control group the activity level is simply recorded, and no further processing occurs.

In the intervention group the data from the first day is used to establish baseline activity levels for each epoch. Daily activity is calculated in 2-hour periods (with one 1-hour period). Each day, the watch calculates a goal for the patient for each period, the goal being 5% higher than the activity recorded in the same period 24 hours earlier.

To gather data from the ZGPAX S8 a custom app was written, based around work by Amor et al. [14] and Ahanathapillai et al. [15], which has been used in previous work looking at physical activity monitoring, among other parameters, in older people. The app records data from the ZGPAX S8 tri-axial accelerometer and processes it to extract a measure of physical activity, the activity score [16]. A slightly modified activity score has been shown to correlate well with energy expenditure, measured in kilocalories per minute (kcal/min), in results obtained from a comparison of activity score against energy expenditure obtained from whole-room calorimetry [17].

Calculation of the activity score [16] and modification of the activity score [14] have been fully described previously; only brief details are repeated here. Raw acceleration data are recorded from the CHMON system in three orthogonal axes, X, Y and Z, at 16.6 Hz using a 30-sec on, 120-sec off interval-sampling regime. The 30-sec-on period is subsequently referred to as an epoch. Sampling was carried out in this manner to preserve battery life on the device whilst endeavoring to capture as much of the patient’s activity as possible. The intention is to compare *relative* levels of activity from day to day, over the treatment period, and between groups. It is not intended to measure actual activity. Using an accelerometer located on the wrist of the unaffected arm, and a sampling procedure covering 20% of the time should allow this without risking systematic bias.

Subsequent data processing of the raw data happens within the watch using inbuilt software in three stages. In the first stage, baseline smoothing is used to remove some of the noise from the signal that is caused by analogue to digital conversion. This has the effect of significantly reducing noise when the CHMON is stationary and where noise therefore is the overriding component in the signal. This is performed on all three axes independently.

In the second stage, the root mean squared combination of the X, Y and Z axes is calculated. The effect of gravity on the accelerometer data (causing a continuous 1 g reading towards the ground) is removed by subtracting the mean of the signal with a 3-sec sliding window. From these data, an assessment of the CHMON worn-state, either worn or not worn, is carried out, which looks to see if the data show any movement and if they do not, the CHMON system is assumed to be not worn and no further processing is applied.

In the final stage, the mean value of the data over the epoch is calculated and adjusted to increase the variance at the lower end of the value range. The value is left unrounded and is used as the activity score [12].

Activity score data from each epoch are added to a tally of activity score for the current time period and these data are used, in the feedback group only, both to provide feedback to and to set goals for the patient. Goals for a day are set to be 5% more in each period that the sum activity score obtained in the corresponding period the previous day.

**Fig. 3:**
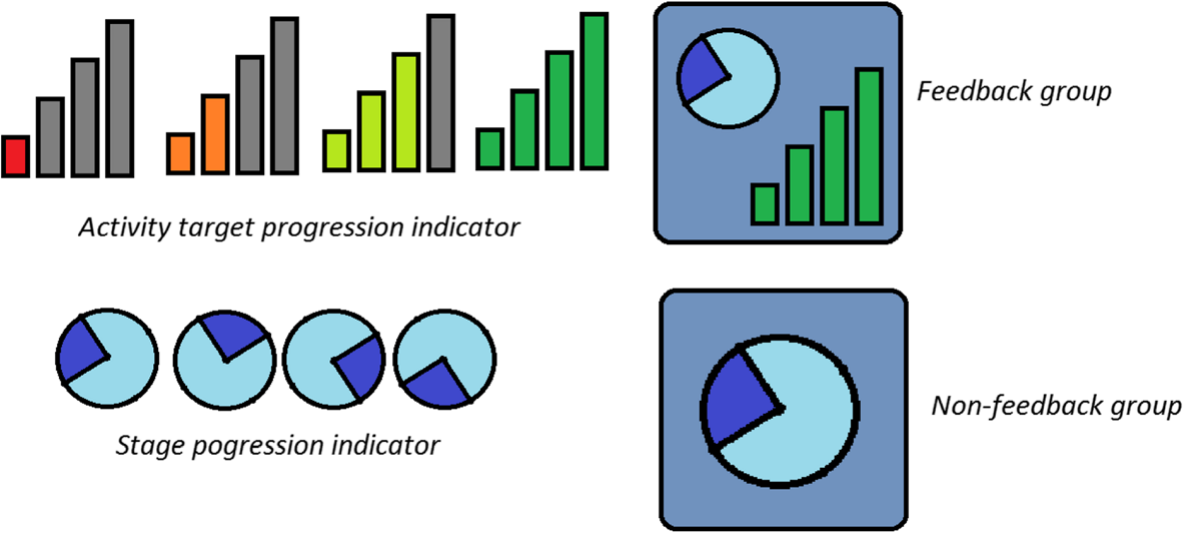
Progression of activity completion (top row) and time epoch (below) on the watch face. Note, only the intervention group will see activity feedback

There are two parts to the user interface; the clock icon and the activity tracker (Fig. 3). The clock icon shows the participant which time period they are currently in. The activity tracker shows how close the patient is to meeting their activity target for the day. All patients will see the clock icon.

**Fig. 4:**
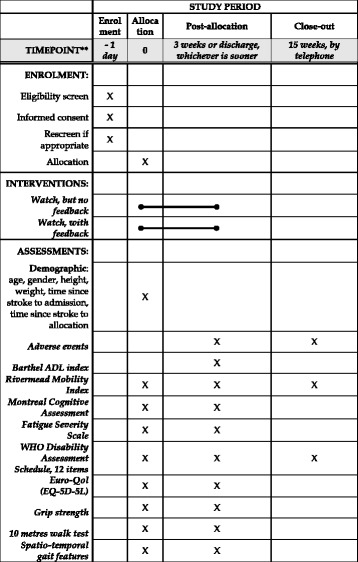
Standard protocol items: recommendation for interventional trials (SPIRIT) figure of study timing and activities. ADL, activities of daily living; WHO, World Health Organization

Each bar of the activity tracker fills as the patient meets a particular percentage of their target activity in the particular epoch. These are set to 1/3, 2/3 and 3/3 of the target such that the next bar (from red to green) will light up when the patient passes these markers. The full green bar lights up when the patient has met or exceeded their target. (See Fig. 4) The feedback page is only shown to the feedback group during the active time periods. At all other times, and at all times for the non-feedback group, the screen simply shows a larger version of the clock icon.

The first day will serve as a baseline measure, in which none of the participants will receive feedback on their activity levels. Thereafter each morning, an investigator will provide the participant with a Smart watch to be worn on the unaffected side and will remind the participant about the purpose of the watch; either to record activity (control group) or to provide feedback on activity (intervention group). Each evening an investigator will see the person, check that there have been no unexpected events or problems such as falls, or other adverse events, and then take the watch away so that data can be downloaded, and the watch recharged for use the next day.

In order to ensure that all researchers administer the intervention in a consistent manner, we will host a series of in-service training sessions on the use of the Smart watch. These in-services will focus specifically on the setup, data storage, and output from the CHMON system.

The control group will wear the same watch, and be seen each day by the researcher to collect the watch. In this way we will control for additional attention, and to an extent for any expectations generated by involvement in a trial and wearing a watch.

#### Data collected

The assessment schedule is shown in Fig. 4. The researchers will collect the following demographic and medical information from clinical notes on admission to rehabilitation: age, sex, height, weight and dates of stroke onset and admission. Moreover at each contact the research doctors will ask the patient about any problems with the watch, and will look out for any observable adverse effects such as a skin rash. The primary data will be the daily activity counts generated by the Smart watch and downloaded daily by the researcher.

Data will be collected by investigators trained in the use of the measures, using Chinese versions of the measures where relevant. The investigator will not know which group the participant is in. The following clinical data will be collected at baseline, end of intervention or at 3 weeks, and some will be collected by phone at 3 months (see Fig. 4):

- The Barthel Activities of Daily Living (ADL) Index (BI) [18], a 10-item scale that assesses the ability of an individual with a neuromuscular or musculoskeletal disorder to care for him/herself.
- The Rivermead Mobility Index (RMI) [19], a 15-item rating scale (14-self-reported items and 1 direct observation item) that assesses functional mobility following stroke (e.g. gait, balance, and transfers).
- The Montreal Cognitive Assessment (MOCA) [20] (Mandarin version) [21, 22], a 16-item scale that assesses the cognitive abilities designed to detect mild cognitive dysfunction.
- The Fatigue Severity Scale (FSS) [23–25], a 9-item scale that measures the severity of fatigue and its effect on a person’s activities and lifestyle.
- The World Health Organization 12 item Disability Assessment Schedule (WHODAS) [26], a questionnaire that assesses six domains of day-to-day functioning and provides an overall disability score based on this.
- The Euro-QOL (EQ-5D-3L) [27, 28], a questionnaire that provides a simple measure of health for clinical and economic appraisal. This questionnaire measures the dimension of mobility, self-care, usual activities, pain/discomfort and anxiety/depression.
- Grip strength [29] provides a quantitative and objective measure of isometric muscular strength of the hand and forearm.
- The 10-m walk test (10MWT) [30] assesses walking speed over 10 m.
- Spatio-temporal gait features at self-selected walking speed measured during a 10MWT using an inertial sensor (LPMS-B, Life Performance Research, Japan) on the lower trunk. Participants will be instructed to walk twice along a 10-m walkway at their normal pace with a walking aid or the support of a researcher. Walking speed, cadence, step length and symmetry of spatio-temporal measures will be calculated [31].

The outcome measure on which the intervention study is powered is change in activity scores as measured by a tri-axial accelerometer from a Smart watch.

Patients with stroke tend to be discharged from in-patient rehabilitation at the Second Affiliated Hospital of Anhui University of Traditional Chinese Medicine after 2–3 weeks. Therefore, the length of the inpatient intervention phase will vary between participants but will be no more than 3 weeks. Activity score (i.e. counts) are summarised at five time points (i.e. at 2-hour increments) throughout the day. Summary analysis, from each time point and from the whole day will be used in the analysis.

#### Data handling and analysis

The data will be collected by researchers on the ward using prepared forms. The forms will be given to the research office where they will be entered into an Excel spreadsheet database. The paper forms will be stored in secure, locked files. De-identified and encrypted data will then be sent electronically on a weekly basis to Oxford Brookes University where they will be checked (a) for obvious errors (e.g. data outside the possible range) and (b) for missing data. If needed, the research centre in Hefei will be contacted to clarify apparent errors and to confirm whether missing data are truly absent. The electronic data will be stored in a secure database at Oxford Brookes University, but all investigators will have free access to all electronic data.

The following measures will be compared between the two intervention groups: Barthel ADL index, Rivermead Mobility Index, Fatigue Severity Scale, Montreal Cognitive Assessment, WHO Disability Assessment Scale, EuroQol 5D-5L, and length of inpatient stay. Data will be analysed based on the intention-to-treat principle.

Descriptive statistics will be calculated for demographic characteristics and compliance data. The independent samples or the chi-square (X^2^) test will be used to assess differences between group means and frequencies at baseline, the test being chosen according to data type. Measures that differ significantly between the two groups will be included as covariates in statistical models comparing interventions. The distribution of data will be analysed.

SPSS v23 will be used to investigate progression within groups by regression analysis. The frequency that each person achieves their activity goal, as set automatically by the watch, will be explored within the intervention groups at discharge from inpatient rehabilitation.

For outcome data, the linear mixed models (LMM) procedure in SAS 9.4 will be used to determine the mean changes in measures, as response variables, according to two intervention regimes (intervention and control) and three repeated measurements, using baseline as a covariate. Distribution of the data will be checked according to various methods of analysis, which are employed at different stages of the study. The analysis of outcome, which will be based on LMM, assumes a Gaussian distribution that can be checked and tested by standard methods. However, we also note that LMM is, in general, quite robust to violation of the distributional assumptions.

This model treats all factors as categorical, and so different types of data can be used. Further and based on the differences in least squares (marginal) means between the two groups (intervention vs control), provided by LMM analysis, power, effect size (Cohen’s d) and their 95% non-central confidence limits will be calculated. Analysis will be used for each outcome measure. The “group” term is the intervention group; the “time” term is the assessment time point. The group-by-time interaction effect will reveal if there is a greater change over time in one group compared with the other.

For a sample size estimate, we used a conservative estimate of the standardised effect size of change in physical activity levels from the initial assessment to discharge from inpatient rehabilitation (i.e. the first phase of the study) as our primary outcome of interest. Our pilot work suggests an effect size of 0.12, and for alpha 0.05 and power 0.95 for repeated measures with three measurement points, we expect a sample of 182 to be adequately powered. From pilot work we expect 10% to drop out, and so we propose to recruit 200 patients.

## Discussion

This study investigates whether setting personalized activity goals derived from and coupled with personal and early feedback on the amount of activity undertaken throughout each day can enhance self-generated activity levels, and whether any increase is associated with improved functional outcomes. The study design integrates the novel intervention into current practice which ensures that the results should be generally applicable. This discussion will cover acknowledged weaknesses and limitations, some arising from technological and financial limitations and others, which follow on from new information published after the protocol was finalized in July 2015. The discussion will also cover strengths and potential outputs in terms of new knowledge.

Technological and financial limitations underlie many of the weaknesses. The watch is only worn during the five working days and only for nine working hours each day. Ideally the watch would record activity and provide feedback throughout the whole time a patient is awake, including weekends and evenings. This limitation reflects constraints associated both with the watch and with the setting. Data storage is limited, which means that only 20% of the activity can be recorded. In addition, download of data is required each day, which requires a trained person, who is only available in working hours. Third, the watch needs charging each day, again requiring a trained person. With advances in technology these constraints should be overcome.

The goal-setting algorithm designed to make allowance for natural daily routines such as time of getting up, meal times etc. is rigid, with fixed 2-hour slots. The increment chosen - 5% - was arbitrary and further studies may be needed to discover whether there is any better increment. The increment may need to vary over the course of recovery. In the early days and weeks quite large increases will occur naturally, but over time the natural rate of change drops and a stable increment might be more applicable after the first 3 months. The visual feedback display seems appropriate, but again there may be better designs; there is always a compromise between detail and simplicity. Recent research suggests that feedback coupled with goal setting can be effective [11], and so an improvement in technology coupled with increasing knowledge may make the goal setting and feedback system more effective.

A third limiting factor arises from the funding and timing of inpatient rehabilitation in Hefei, which starts quite late after stroke and is limited to 2 or 3 weeks of inpatient rehabilitation, limiting the time the intervention can be applied. By the time a person is admitted, they have already established a pattern of behaviour. Also, it may take at least 1 week to become familiar with the device and its purpose and, because changing behaviour takes time, the intervention may be too short to lead to any detectable difference between the groups. It was not possible to provide the watch for use at home, mainly for practical reasons (daily data downloads and recharging). Ideally the intervention should be continued over many weeks.

The research funding was limited. The research data were collected by the ward doctors, who were specifically trained and collected the clinical data as part of their normal work. However, this limited the options of using more fixed procedures, for example when checking cognitive ability. This will not cause any bias, but may slightly limit generalizability.

Last, it has been assumed that increasing general, non-specific activity levels will lead to specific changes in mobility and independence is activities. This assumption is reasonable, but the strength of the relationship is unknown and a much larger study may be needed to show any actual benefit.

One strength is that, according to our calculations, the study is adequately powered; we will recruit about 200 patients. Second, the study is embedded with daily clinical routines in a busy hospital, such that if the intervention is effective, it should be easy to continue the intervention.

If this study does not show an effect, there will still be worthwhile knowledge generated. The study will give an estimate of variance in the measures used, which will help in future power calculations. It will discover whether a short episode of feedback on activity set against goals might have an effect. Failure to find an effect should not be taken as proving the intervention is ineffective, given the limitations identified. The study will give information about the feasibility of collecting data in a trial set within a standard rehabilitation programme in China (Additional file 1).

## Trial status

At the time of original submission (20 February 2017) recruitment in the trial had started and 88 patients had been recruited. Recruitment ended with 160 patients on 16 June 2017 with final follow up on 16 September 2017.

## Additional file

Additional file 1: SPIRIT 2013 Checklist: Recommended items to address in a clinical trial protocol and related documents. (DOCX 118 kb)
